# Traumatic Dental Injury—An Enigma for Adolescents: A Series of Case Reports

**DOI:** 10.1155/2012/756526

**Published:** 2012-11-07

**Authors:** Akash Krishna, Manjunath Hampanna Malur, D. V. Swapna, Shiny Benjamin, Chris A. Deepak

**Affiliations:** ^1^Department of Conservative Dentistry and Endodontics, Peoples College of Dental Sciences and Research Centre, Bhopal 462037, India; ^2^Dayanand Sagar College of Dental Sciences, Karnataka, Bangalore 560078, India; ^3^Department of Endodontics, Vydehi Dental College, Karnataka, Bangalore 560066, India

## Abstract

Coronal fractures of permanent dentition are the most frequent type of dental injury. Fractured anterior teeth are usually treated with conventional post and core and crown techniques, after being treated endodontically. If the original tooth fragment is retained following fracture, the natural tooth structures can be reattached using adhesive protocols. Fiber-reinforced post makes the reattachment of the crown esthetically possible with minimal preparation and reduces the possibility of tooth fracture during function. This paper presents the therapeutic approach of reattachment of crown fragment to the tooth at the cervical and middle third levels.

## 1. Introduction

Fracture of anterior teeth by trauma is the most frequent type of injury in the permanent dentition, especially among children and adolescent affecting up to 25% of this patient population [1]. If the fracture also exposes the dental pulp, the injury is defined as a complicated crown fracture or Class 3 fracture (Ellis and Davey classification) [2]. The incidence of complicated crown fractures ranges from 2% to 13% of all dental injuries and the most commonly involved teeth are the maxillary central incisors [3]. Trauma in children and adolescents require greater attention due to the physical and emotional characteristics of both the patient and family alike [4]. Dentists are confronted with managing dental trauma and restoring fractured teeth on a regular basis. Techniques that speed and simplify treatment, restore aesthetics, and improve long term success rates are therefore of potential value and should be considered [1]. Traditionally complicated crown fractures have been restored with conventional post-core and crown techniques after endodontic treatment. Several factors influences the management of coronal tooth fractures including extent of fracture, pattern of fracture and the restorability of the tooth, presence or absence of the fractured tooth fragment, occlusion, and esthetics [5].

Despite the recent advances in adhesive materials and restorative technique there is no restorative material that can reproduce the esthetic and functional needs as much as the natural dental structures [6]. Various treatment modalities have been proposed for the treatment of coronal fracture. One of the options for managing this clinical situation, especially when there is no or minimal violation of the biological width, is the reattachment of dental fragment [7]. Chosack and Eidelman described for the first time the reattachment of tooth fragment after trauma of 12-year-old child. At present, reattachment of fractured tooth fragments should be the first choice to restore fractured teeth [8]. The advantage of reattachment of fractured fragments include immediate esthetics, more reliable outline form, possibility of maintaining the occlusal function, absence of differential wear, lowered economic burden, and excellent time resource management [9].

Resin-based restorative materials are frequently used in restoration of the fractured teeth. Because of the poor mechanical resistance of these materials, different approaches developed to strengthen the composite resin, such as fiber posts [10]. Tooth-colored fiber posts have several advantages, such as esthetic, bond to tooth structure, and having a modulus of elasticity similar to that of dentin, but still require dentin preparation to fit into the canal [11].

 A crown-root fracture involving the biologic width needs to be approached carefully, as placing the margins within the biologic width frequently leads to gingival inflammation, clinical attachment loss, and bone loss [12]. However, in the case of tooth fractures where a juxtaposition of the fragment with the tooth shows that the margins of each fit well against one another and no interfragmentary space is present, an adhesive technique may be considered as an alternative approach [13]. The aim of this paper was to report and discuss the management of complicated crown fractures in three clinical situations.

## 2. Case 1

**Figure 1:**
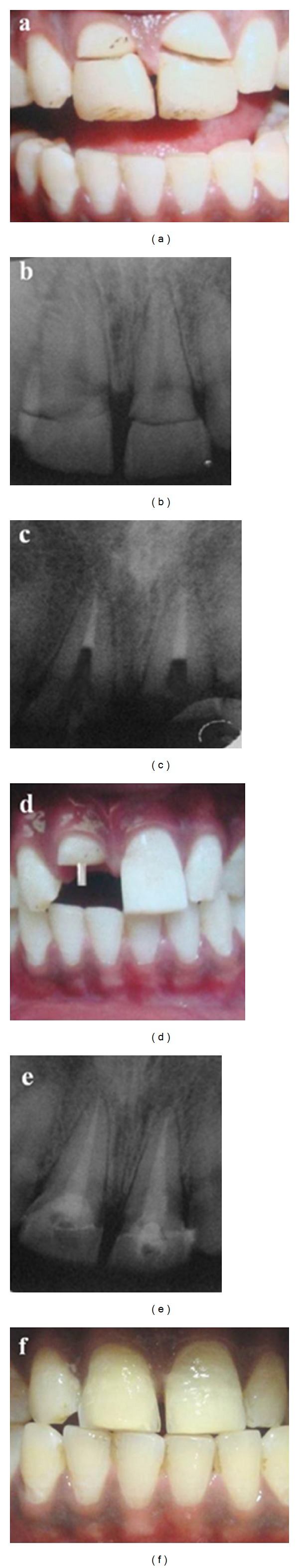
Reattachment of fractured tooth segments using a prefabricated fiber post. (a) Clinical appearance of the patient, (b) preoperative radiograph, (c) sectional obturation of the root canals, (d) fiber post luted in the root canal with 2 mm of the retentive part of the post outside the chamber, (e) postoperative radiograph, and (f) clinical view after segments reattachment.

A 23-year-old male patient was referred to the Department of Conservative Dentistry and Endodontics, with the complaint of fractured maxillary central incisors because of road traffic accident. The patient's medical history was noncontributory. Initial examination revealed the horizontal fracture on teeth no. 11 and 21, and associated pulp exposure (Figure 1(a)). A diagnosis of Ellis class 3 fracture (complicated crown fracture) was made. On clinical examination the fracture lines were extended obliquely, from labial to lingual direction. The fragments were still attached by soft tissues at the palatal aspect. Radiographic examination showed the horizontal fracture line at cervical third of both the crowns, and no root fracture (Figure 1(b)). The patient expressed the desire to maintain teeth and restore it. A detailed explanation about the treatment plan was given to the patient, including root canal treatment and then reattachment of the crowns by using fiber-reinforced posts. The treatment plan was accepted by the patient.

After local anesthesia, the coronal fragments were separated with minimum force and were stored in saline to prevent dehydration. Single visit root canal treatments were carried out on both the teeth, and only the apical thirds were obturated by gutta percha with sectional obturation technique (Figure 1(c)). The post spaces were prepared with corresponding drills to receive the fiber reinforced post (C0601 no. 1, Easy post, Dentsply, Maillefer). The posts were checked in the canal for proper length and adaptation. After isolation, canal walls of both the teeth were etched with 37% phosphoric acid for 15 seconds, rinsed, and dried with paper points. Bonding agent (Adper single bond 2, 3M ESPE) was then applied to the root canal walls with a microbrush in two coats and gently air dried followed by light curing for 15 seconds. Thin layer of dual cure composite resin cement (3M_ RelyX_ Adhesive Resin Cement, 3M ESPE) was applied, and the post was cemented into the canal such that 2 mm of its coronal portion was outside the chamber (Figure 1(d)). The fractured segments were cleaned to remove pulpal remnants. Box-like preparations were made in the pulp chamber of fractured segment which corresponded to the retentive part of extruding post. The remaining part of tooth structure, the posts, and the fractured segments were etched and two coats of dentine bonding agent (Single bond 3M ESPE) were applied and cured for 10 seconds. Both fractured segments were reattached with dual cure composite resin cement (3M_ RelyX_ Adhesive Resin Cement, 3M ESPE). Margins were light cured for 40 seconds from various directions. Silicon-based polishing discs (Soft-Lex, 3M ESPE) were used in decreasing coarseness to polish marginal areas (Figures 1(e) and 1(f)). At 12-month-followup, results were satisfactory in terms of fragment stabilization and esthetics.

## 3. Case 2

**Figure 2:**
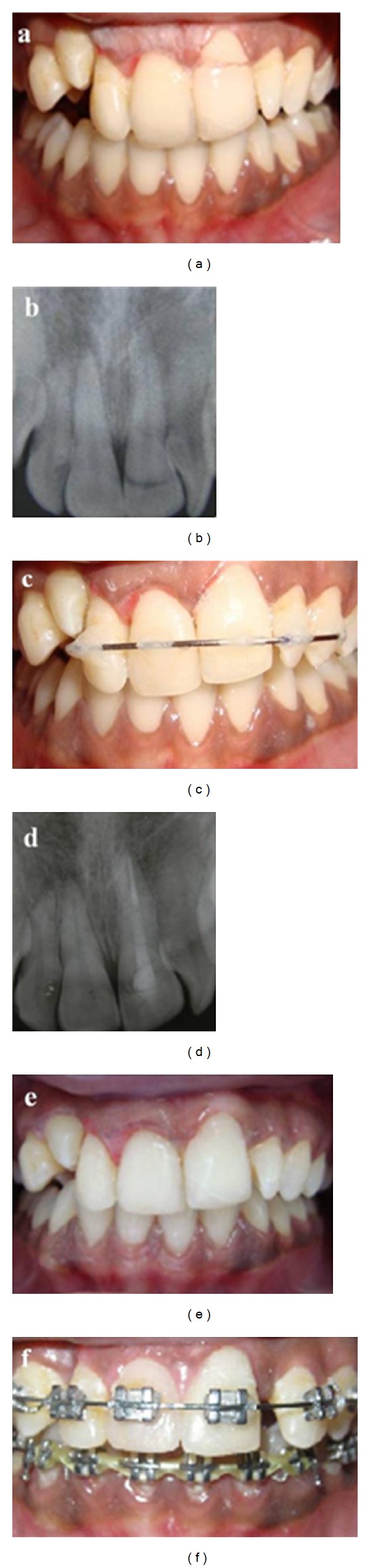
Reattachment of fractured tooth segments and reinforcing the attachment by pre-fabricated fiber post. (a) Clinical appearance of the patient, (b) preoperative radiograph, (c) reattachment of fractured tooth segment, and splinting with stainless steel wire and light cure composite resin, (d) radiograph showing fiber post reinforcing the attachment, (e) clinical view after removal of the splint, and (f) clinical view showing orthodontic treatment in progress.

A 21-year-old female patient attended the Department of Conservative dentistry and Endodontics, 2 days after an automobile accident. The patient had multiple lacerations on the face and fractured crown. After the general medical, dental, and traumatic incident histories were reviewed, clinical and radiographic examinations were conducted. A complicated crown fracture was observed in relation to left maxillary central incisor, tooth no. 21 (Figure 2(a)). The crown fragment was mobile but still attached on the palatal surface by the junctional epithelium and connective tissue. The patient was in severe pain as a result of a large pulp exposure. Adjacent teeth had slight mobility with redness at the marginal gingiva suggestive of some tooth displacement. Radiographic examination revealed horizontal fracture line at cervical third of the crown in relation to tooth no. 21, and no root fracture (Figure 2(b)). A detailed explanation about the treatment plan was given to the patient, including emergency root canal treatment, reattachment of the fractured crown, splinting of the anterior teeth, followed by reinforcing the attachment by fiber reinforced post. The treatment plan was accepted by the patient and informed consent was obtained.

Local anesthesia was administered and fractured segment was gently removed and stored in saline. One visit root canal treatment was performed. The fractured segment was reattached as described in case report 1. Since adjacent teeth had mobility, they were splinted with 19-gauge stainless steel wire and light cure composite resin (Figure 2(c)). At the following visit the canal was prepared to receive the fiber reinforced post, by removing the obturated gutta percha till apical third. The canal walls were etched, rinsed, dried, and treated with bonding agent and fiber-reinforced post was cemented into the canal as described in case report 1 (Figure 2(d)). The patient was advised to report after a week for the removal of the splint (Figure 2(e)). Patient was recalled after 1 month for followup. The patient had no signs or symptoms and wanted to go for orthodontic consultation. One-year followup showed stabilization of the fragment, and orthodontic treatment in progress (Figure 2(f)).

## 4. Case 3

**Figure 3:**
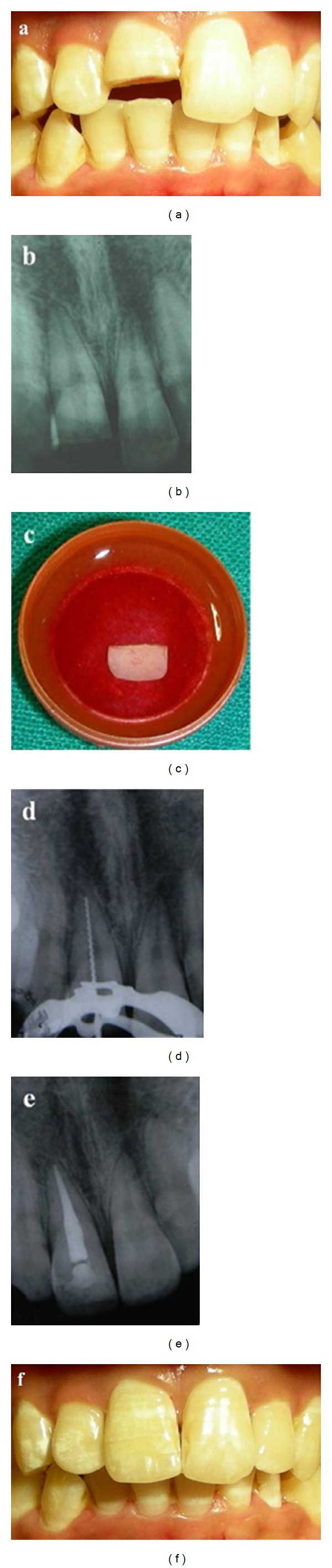
Reattachment of fractured tooth segment. (a) Clinical appearance of the patient, (b) preoperative radiograph, (c) fractured crown segment bought by the patient, (d) working length radiograph, (e) postoperative radiograph, and (f) clinical view after segment reattachment.

A 14-year-old patient reported to the Department of Conservative dentistry and Endodontics, 2 days after sustaining crown fracture to his maxillary right central incisor during sports activities. After the general medical, dental and traumatic incident histories were reviewed and clinical and radiographic examinations were conducted. Clinical examination revealed a fracture of the middle third of the crown, exposing the pulp with tooth no. 11 (Figure 3(a)). The remaining maxillary and mandibular anterior teeth were intact. Radiographic examination revealed horizontal fracture at middle third of the crown, an intact periodontal ligament space, and complete root formation with no root fracture (Figure 3(b)). The patient had brought the displaced tooth segment in water (Figure 3(c)). The fractured segment could be closely adapted to the remaining crown structure. Hence, one-visit root canal treatment followed by reattachment of the fractured segment was planned. The procedure was explained to the patient and her parents and informed consent was obtained. Following local anaesthesia root canal treatment was performed (Figure 3(d)). The fractured segment was stored in normal saline throughout and then cleaned to remove pulpal remnants. The remaining tooth structure, chamber, and the fractured segment were etched with 35% phosphoric acid (3M ESPE, USA) for 15 seconds. The etchant was washed for 10 seconds and the cavity was dried with a gentle blast of air. Caution was taken not to desiccate the surfaces. Two coats of dentine bonding agent (Single bond 3M ESPE) were applied at an interval of 10 seconds and cured for 10 seconds. The segment was reattached with dual cure composite resin cement (3M_ RelyX_ Adhesive Resin Cement, 3M ESPE) (Figure 3(e)). All margins were light cured for 40 s and then polished using diamond stones and a composite polishing kit (Shofu Co., Kyoto, Japan) (Figure 3(f)). At 12-month-followup, the tooth was symptom-free and the crown was aesthetically satisfactory.

## 5. Discussion

Loss of tooth tissue in the anterior region in a young patient may create severe aesthetic and emotional problems [14]. Functional, aesthetic, and biologic restoration of the fractured incisors often presents a daunting clinical challenge. Various treatment approaches have been indicated for fractured teeth including, fragment removal followed by restoration [15]; fragment reattachment [16]; gingivectomy and osteotomy (crown lengthening) [16]; orthodontic extrusion with/without gingivoplasty [15, 16]; forced surgical extrusion [15, 16]; vital root submergence [16]; extraction followed by surgical implants [16] or fixed partial denture [17].

Reattachment of the crown fragment to a fractured tooth influences esthetic by retaining natural translucency and surface texture and is the first choice for crown fractures of anterior teeth. Once the original fragment is reattached, the natural appearance will be restored instantly. Also, this procedure is relatively simple, atraumatic, and inexpensive. This technique requires only a thin layer of composite resin and restores the original form and color of the tooth that often provides the best aesthetic result. Several case reports show that even subgingival tooth fractures can be restored successfully [13]. Studies have shown that 85% of traumatized incisors fracture line runs obliquely from labial to lingual aspects with the fracture line proceeding in an apical direction. Hence, such type of unfavorable fracture restoration would have low resistance to labially applied forces, like a traumatic force itself, but may have higher resistance to horizontal forces which occur with incising or tearing food [1].

In our cases we have used reattachment technique of the autogenous tooth fragment to the crown, with or without fiber reinforced post. Although the use of pre-fabricated post does not mechanically strength the endodontically treated teeth, but it helps in retention of the coronal restoration [18].

Fiber reinforced post has demonstrated negligible root fracture. In addition, the fiber reinforced post can be used with minimal preparation because it uses the undercuts and surface irregularities to increase the surface area for bonding. Thus, it reduces the possibility of tooth fracture during function or traumatic injury [19]. The use of dentine adhesive with high demonstrable bond strength and the use of composites resins optimize esthetics. However, absence of traumatic occlusion should be confirmed for the long term clinical success. In Case 2, it is remarkable to notice that reattachment of the fractured crown and reinforcing the attachment by fiber reinforced post permitted the tooth to bear orthodontic forces and desirable tooth movements. Besides long term followsups are necessary. Recall examination after 1-, 6-, and 12-month intervals permits observation and provides feedback about the outcome of the treatment protocol.

## 6. Conclusion

The rule of nature is to conserve as much of tooth structure as possible. Preservation of the remaining natural and healthy tooth structure will have a positive psychological impact on the patient. With the advancements in the chemistry of dentine adhesives and composite resins having remarkable bond strength, the success rate has been enhanced. Reattachment of fractured fragment and reinforcing it with fiber post is a viable technique that restores function and esthetics with a conservative approach. Although unsupported by the laboratory studies and by many clinical trials, results of multicenter clinical studies have indicated a more favorable prognosis when the dentist is well equipped with the knowledge about the clinical situation, and advancements in the material sciences.
